# Node2Vec-DGI-EL: a hierarchical graph representation learning model for ingredient–disease association prediction

**DOI:** 10.1093/bioadv/vbaf216

**Published:** 2025-12-10

**Authors:** Leifeng Zhang, Xin Dong, Shuaibing Jia, Jianhua Zhang

**Affiliations:** Medical Engineering Technology and Data Mining Institute, Zhengzhou University, Henan 450000, China; Medical Engineering Technology and Data Mining Institute, Zhengzhou University, Henan 450000, China; Faculty of Innovation Engineering, Macau University of Science and Technology, Taipa, Macao 999078, China; School of Pharmacy, Macau University of Science and Technology, Taipa, Macao 999078, China; Medical Engineering Technology and Data Mining Institute, Zhengzhou University, Henan 450000, China; Medical Engineering Technology and Data Mining Institute, Zhengzhou University, Henan 450000, China; School of Pharmacy, Macau University of Science and Technology, Taipa, Macao 999078, China

## Abstract

**Motivation:**

Traditional Chinese medicine, as an essential component of traditional medicine, contains active ingredients that serve as a crucial source for modern drug development. To explore the potential application value of traditional Chinese medicine ingredients, this study utilizes the complex network formed between herbs, ingredients, targets, and diseases, and proposes an ingredient–disease association prediction model (Node2Vec-DGI-EL) based on hierarchical graph representation learning. The model first utilized Node2Vec to extract node embedding vectors, serving as the initial features for the network nodes. Then, DGI was applied to further refine the node representations, enhancing the model’s expressive power. Finally, an ensemble learning method was integrated to further improve prediction performance.

**Results:**

The proposed model significantly outperformed existing methods, achieving an AUC of 0.9987 and an AUPR of 0.9545. Case studies further validated the reliability of the model’s predictive results. Specifically, triptonide exhibited a binding energy of −9.62 kcal/mol with PGR, a core target of hypertensive retinopathy, while methyl ursolate showed a binding energy of −9.71 kcal/mol with NFE2L2, a core target of colorectal cancer. The Node2Vec-DGI-EL model focuses on traditional Chinese medicine datasets, effectively predicting ingredient–disease associations. It demonstrates significant application value and can assist in drug repositioning and novel drug development.

**Availability and implementation:**

The code and data are available at https://github.com/wayfarer569/Node2Vec-DGI-EL.

## 1 Introduction

As natural medicines, ingredients of traditional Chinese medicine (TCM) are an important source for modern drug development, containing great therapeutic potential and research value. However, traditional applications of TCM are mostly based on empirical knowledge and historical practices, which has limited the exploration of these ingredients for treating other diseases, thereby restricting their broader application and development in modern medicine. The rise of network pharmacology has provided a new perspective for TCM research by constructing multilayered herb-disease networks to systematically reveal the material basis of efficacy and molecular mechanisms (Hopkins 2008). In recent years, the rapid development of artificial intelligence (AI) and knowledge graphs has further accelerated the advancement of TCM research based on biological networks and big data (Zhang *et al.* 2023). Neural network algorithms have emerged as powerful tools for predicting drug-disease potential associations, accelerating the processes of drug discovery and drug repositioning (Wan *et al.* 2019). Therefore, we can explore potential associations between ingredients and diseases through neural network algorithms, revealing the therapeutic potential of ingredients in other diseases.

In drug–disease association prediction research, Gottlieb *et al.* (2011) employed a logistic regression framework to evaluate and rank the evidence of associations between query pairs and known drug-disease relationships based on drug-drug and disease-disease similarities. Kim *et al.* (2019) employed machine learning techniques to predict drug-disease associations by utilizing four types of drug-drug similarities and three types of disease-disease similarities. Luo *et al.* (2018b) adopted a heterogeneous network and random walk model for candidate disease prediction. Luo *et al.* (2018a) developed the DRRS system, which employs the SVT algorithm to fill in the drug-disease network for drug repositioning prediction. Zhang *et al.* (2018) proposed the SCMFDD algorithm, which integrates drug-disease associations and feature information to reveal latent relationships through low-rank space projection, incorporating drug similarity and disease semantic similarity as constraints. Yu *et al.* (2021) proposed the LAGCN algorithm, which integrates drug-disease associations, drug-drug similarities, and disease-disease similarities using graph convolution and attention mechanisms to predict new associations. Kang *et al.* (2023) proposed the LBMFF method, which utilizes the BERT model to extract semantic information from literature, combined with graph convolution and attention mechanisms to reveal latent associations between drugs and diseases.

In the study of TCM networks, Wang *et al.* (2019) proposed the HTINet method, which generates node sequences through random walks, combines shallow neural networks to learn low-dimensional vector representations, thus avoiding reliance on node semantic information and enabling herbal target prediction. Subsequently, Duan *et al.* (2024) proposed HTINet2 based on HTINet, incorporating residual GCN and optimizing through the BPR loss function, which enhanced the performance of herbal target prediction.

Existing drug–disease prediction models rely on constructing drug–drug and disease–disease similarity matrices. However, due to the large scale of TCM networks, it is challenging to comprehensively organize the prior knowledge of nodes to build ingredient–ingredient and disease–disease similarity matrices. This makes it difficult for current models to effectively apply in ingredient–disease association prediction. Herbal target prediction models, during the neural network learning phase, focus solely on the associations between herbs and targets, neglecting the interactions with other entities.

To address the above issues, we propose the Node2Vec-DGI-EL model for ingredient–disease association prediction. It combines the Node2Vec (Grover and Leskovec 2016), the Deep Graph Infomax algorithm (DGI) (Veličković *et al.* 2018), and ensemble learning, offering three key advantages. First, the Node2Vec generates node embeddings through biased random walks, focusing on local structural information, which provides high-quality initial features for the subsequent DGI and reduces dependence on prior knowledge. The DGI integrates both local and global information through unsupervised contrastive learning to capture higher-order dependencies, resulting in high-quality node embeddings. Finally, the ensemble learning model trains multiple balanced subsets to mitigate the negative impact of class imbalance on prediction accuracy, thus improving the overall predictive performance of the model.

## 2 Materials and methods

### 2.1 Data collection

This study utilized the Encyclopedia of Traditional Chinese Medicine (ETCM) (Xu *et al.* 2019) to collect association data for four key entities: TCMs, active ingredients, protein targets, and diseases. To enhance the biological relevance of the network, we further integrated protein-protein interaction (PPI) data from the STRING (Szklarczyk *et al.* 2023), applying stringent filtering criteria to retain only high-confidence interactions with a combined score ≥700. The integrated dataset was used to construct a comprehensive Herb-Ingredient-Target-Disease association network (HITD), which encompasses four types of entities (herbs: 402, ingredients: 6994, targets: 16 610, and diseases: 4289) and seven types of associations. The complete network architecture is presented in Table 1.

**Table 1.** HITD network data statistics.

Type	Nodes	Nodes	Edges	Source
Herb-Ingredient	402	6971	10 304	ETCM
Herb-Target	399	1752	50 061	ETCM
Herb-Disease	395	2704	248 845	ETCM
Ingredient-Target	4185	1635	75 112	ETCM
Ingredient-Disease	4029	2661	617 530	ETCM
Disease-Target	4289	4887	73 540	ETCM
Target-Target	16 201	16 201	236 930	STRING
Total			1 312 322	

### 2.2 Model architecture design

**Figure 1.**
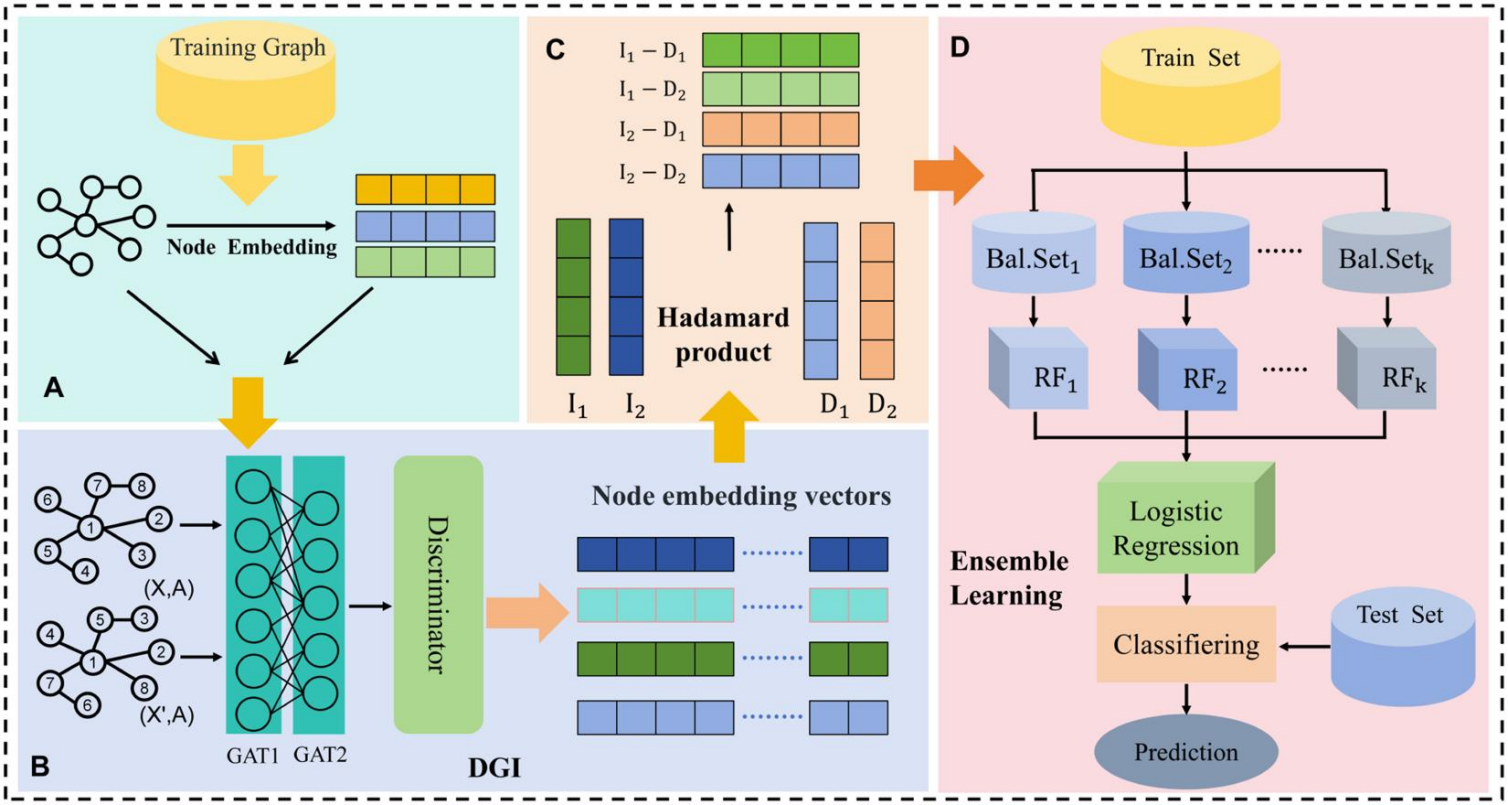
Overall architecture of Node2Vec-DGI-EL. (A) and (B) The node feature extraction modules. (C) The node pair feature construction module. (D) The ensemble learning module.

The architecture of the model in this study consists of three modules: the node feature extraction module, the node pair feature construction module, and the ensemble learning module, as shown in Fig. 1.

#### 2.2.1 Node feature extraction modules

This module combined the Node2Vec and the DGI to extract node features from the graph network. The Node2Vec integrates the ideas of depth-first search (DFS) and breadth-first search (BFS) and introduces two parameters: the return parameter *p* and the in-out parameter *q* to control the random walk strategy. It then uses the Skip-Gram model to learn node embedding vectors. The loss function of the Node2Vec is defined as follows:


(1) $$\begin{array}{c} L=\sum_{u\in V}\sum_{v\in N_{S}(u)}\;[\text{log}\;\sigma (z_{u}^{⊤}z_{v})\\ +\sum_{i=1}^{k}E_{v_{i}∼P_{n}(v)}\;\text{log}\;\sigma (-z_{u}^{⊤}z_{v_{i}})] \end{array}$$

where,

- *V* is the set of all nodes in the graph;
-
$N_{S}(u)$ denotes the sampled neighborhood of node *u* obtained via biased random walks;
-
$z_{u}$ and $z_{v}$ are the embedding vectors of nodes *u* and *v*, respectively;
- *k* is the number of negative samples per positive pair.

Based on the initial node features captured from the network by Node2Vec, the DGI was then used to further learn deeper node embedding representations. DGI is an unsupervised graph learning that learns node embeddings by maximizing the mutual information between node representations and global graph representations. The DGI framework mainly includes four aspects. Firstly, we use graph neural networks to encode each node and generate node embeddings. This article chooses Graph Attention Network (GAT) (Veličković *et al.* 2017) to achieve this goal. Second, the node embeddings were aggregated (mean pooling was used in this article) to generate a global graph representation. Then, negative samples were generated by disrupting the original graph’s node feature order. Finally, through the contrastive learning mechanism, the mutual information between node embeddings and global graph representations was maximized, obtaining high-quality node embeddings.

Global graph representation:


(2) $$s=\frac{1}{N}\sum_{i=1}^{N}h_{i}$$

Discriminator:


(3) $$D\left(h_{i},s\right)=\sigma \left(h_{i}^{⊤}Ws\right)$$

The loss function of the DGI is:


(4) $$\begin{array}{c} L=\frac{1}{N+M}(\sum_{i=1}^{N}E_{\left(X,A\right)}\left[\text{log}\;D(h_{i},s)\right]\\ \;+\sum_{j=1}^{M}E_{\left(\tilde{X},\tilde{A}\right)}\left[\text{log}\;\left(1-D({\tilde{h}}_{j},s)\right)\right]) \end{array}$$

where,

- *N* and *M* denote the numbers of positive and negative samples, respectively;
-
(X,A) represents the input feature matrix and adjacency matrix of the original (positive) graph;
-
$\left(\tilde{X},\tilde{A}\right)$ represents the feature and adjacency matrices of a perturbed or generated (negative) graph;
-
$h_{i}$ is the representation of node *i* from the positive graph;
-
${\tilde{h}}_{j}$ is the representation of node *j* from the negative graph;

#### 2.2.2 Node pair feature construction module

To capture the interaction between node pairs, common methods include concatenation, addition, subtraction, and the Hadamard product. Among them, the Hadamard product is widely adopted due to its simplicity and ability to model similarity via element-wise multiplication. In this study, we adopt the Hadamard product to construct node pair features. Given two nodes with embedding vectors $u_{i}$ and $u_{j}$, their node pair feature is computed as:


(5) $$u_{i,j}=u_{i}⊙u_{j}$$

#### 2.2.3 Ensemble learning module

Due to the sparsity of the network, the number of negative samples in the node pair feature vector dataset is larger than the number of positive samples. This class imbalance issue can lead to machine learning models being biased towards the majority class, affecting the accuracy of predictions (Martínez *et al.* 2017). Ensemble learning has shown better performance in improving the classification performance on imbalanced datasets (Khoshgoftaar *et al*. 2015). Therefore, this study adopted the stacking ensemble learning model (Pavlyshenko 2018), as shown in Fig. 1D. First, the negative samples were divided into multiple independent subsets, each having the same number of samples as the positive class, and each negative subset was combined with the positive samples to construct multiple balanced subsets (Balanced Subset, abbreviated as: Bal.Set). Then, for each balanced subset, the Random Forest algorithm (RF) (Breiman 2001) was used as the base learner for training. Finally, the Logistic Regression algorithm was used as the meta-learner to integrate the predictions from the base learners, generating a more robust prediction result.

### 2.3 Model training and evaluation

#### 2.3.1 Data division

To study the potential associations of ingredients and diseases, this study randomly hid 30% of known ingredient–disease node pairs in the complete HITD network, leaving the rest of the structure unchanged for training. By Node2Vec-DGI obtaining the node embedding vector, the hidden edge node pair was calculated as a positive sample (label 1), and negative samples were sampled proportionally from the unconnected ingredient–disease node pairs (label 0) to construct the feature vector dataset. In this dataset, the number of positive samples is 185 259, and the number of negative samples is 8 813 920, with a positive-to-negative sample ratio of ∼1:47.63. The dataset was divided into training and test sets at a 7:3 ratio for training and evaluation of the ensemble learning model.

#### 2.3.2 Evaluation methods

The ingredient–disease association prediction task can be regarded as a typical binary classification problem. This paper employs AUC (Yang *et al.* 2015), AUPR (Zhou 2021), as well as Accuracy, Precision, Recall, and F1 score to comprehensively evaluate the model’s performance. Initially, hyperparameter tuning was performed to analyze the impact of different hyperparameters on model performance and to select the most suitable parameter combination. Subsequently, the model was compared with several existing models, followed by an ablation study to investigate the importance of each module. Finally, robustness experiments were conducted by removing ingredient–disease association edges at varying proportions (from 10% to 100%) to assess the model’s stability under conditions of missing data.

#### 2.3.3 Case study verification method

This study obtained ingredient targets based on the SEA (Keiser *et al.* 2007) and SwissTargetPrediction (Daina *et al.* 2019), and screened disease targets using GeneCards (relevance score > median) (Stelzer *et al.* 2016). Intersection targets were obtained using the Venny tool, and a PPI network was constructed with the STRING. The network was visualized using Cytoscape 3.9.1 (Shannon *et al.* 2003), and combined with the cytoHubba (Chin *et al.* 2014) plugin, the top 10 core targets were screened using the Maximal Clique Centrality (MCC) algorithm. Ingredient and target protein structures were obtained from PubChem (Kim *et al.* 2025) and the PDB (Protein Data Bank Contributors 2019). After conversion to PDBQT format using Open Babel (O’Boyle *et al.* 2011), molecular docking was performed with AutoDock 1.5.7 (Morris *et al*. 2009), and the docking results were finally visualized using PyMOL (DeLano 2002).

## 3 Results

### 3.1 Model selection

This section compares five classic graph representation learning algorithms, including DeepWalk (Perozzi *et al.* 2014), Node2Vec, Struc2vec (Ribeiro *et al.* 2017), LINE (Tang *et al.* 2015), and SDNE (Wang *et al.* 2016), combined with the DGI (Table 2). The results show that Node2Vec-DGI-EL demonstrates the optimal predictive performance, with all indicators outperforming other models, achieving an AUC value of 0.9976 and an AUPR value of 0.9398. Furthermore, for this model, the impact of different embedding dimensions of the Node2Vec on model performance is explored (Table 3). The results indicate that when the Node2Vec uses an embedding dimension of 128, the model’s prediction results are optimal.

**Table 2.** The prediction results of five models.

Model	AUC	AUPR	ACC	Precision	Recall	F1
DeepWalk-DGI-EL	0.9969	0.9329	0.9693	0.6993	0.9744	0.7756
Struc2vec-DGI-EL	0.9972	0.9257	0.9717	0.7089	0.9753	0.7857
LINE-DGI-EL	0.9971	0.9171	0.9694	0.6997	0.9753	0.7762
SDNE-DGI-EL	0.9971	0.9182	0.9718	0.7096	0.9759	0.7865
Node2Vec-DGI-EL	0.9976	0.9398	0.9752	0.7254	0.9777	0.8026

**Table 3.** The prediction results of different embedding dimensions of Node2Vec.

Dimensions	AUC	AUPR	ACC	Precision	Recall	F1
64	0.9954	0.9379	0.9699	0.7124	0.9732	0.7889
128	0.9976	0.9398	0.9752	0.7254	0.9777	0.8026
192	0.9959	0.9382	0.9691	0.7175	0.9723	0.7842
256	0.9965	0.9391	0.9706	0.7169	0.9726	0.7928
320	0.9954	0.9370	0.9709	0.7185	0.9723	0.7941

### 3.2 Parameter tuning

**Figure 2.**
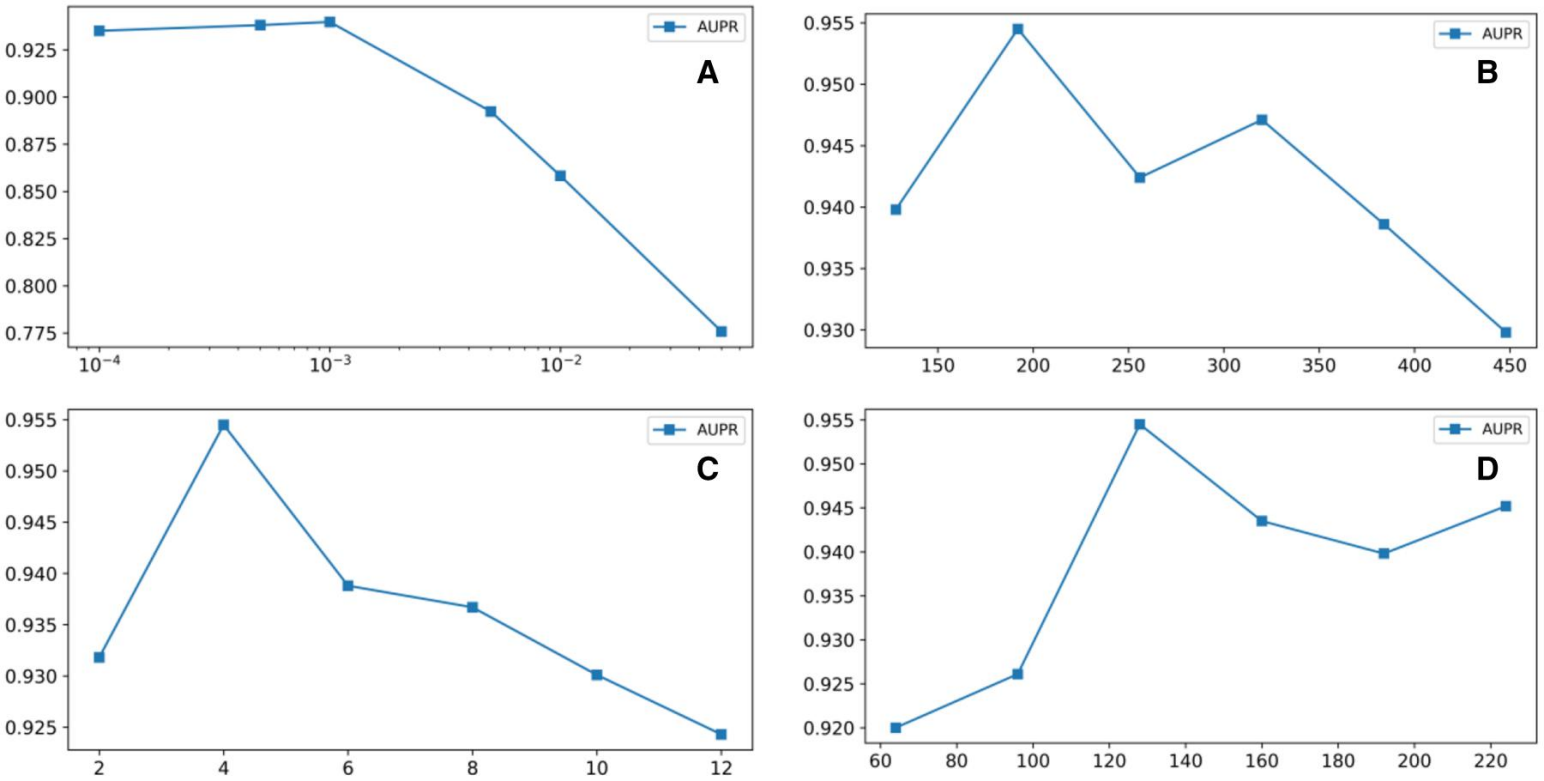
Impact of hyperparameter configurations on model performance. (A) Learning rate. (B) Hidden layer dimension. (C) Number of attention heads. (D) Output dimension.

Based on the 128-dimensional embeddings from the Node2Vec, this section analyzes four hyperparameters of the DGI: learning rate, output dimension, hidden layer dimension, and the number of attention heads. The impact of each hyperparameter within different parameter ranges on model performance was assessed using AUPR as the evaluation metric. The experimental configurations are detailed in Table 4 and the corresponding results are presented in Fig. 2. The results show that the optimal parameters of the DGI are as follows: the learning rate is 0.001; the hidden layer dimension is 192; the output layer dimension is 128; the attention head count is 4.

**Table 4.** Hyperparameter search space.

Hyperparameters	Tested values
LR	0.0001, 0.0005, 0.001, 0.005, 0.01, 0.05
Hidden Dim	128, 192, 256, 320, 384, 448
Output Dim	64, 96, 128, 160, 192, 224
Attention Heads	2, 4, 6, 8, 10, 12

### 3.3 Comparison with baseline method

**Figure 3.**
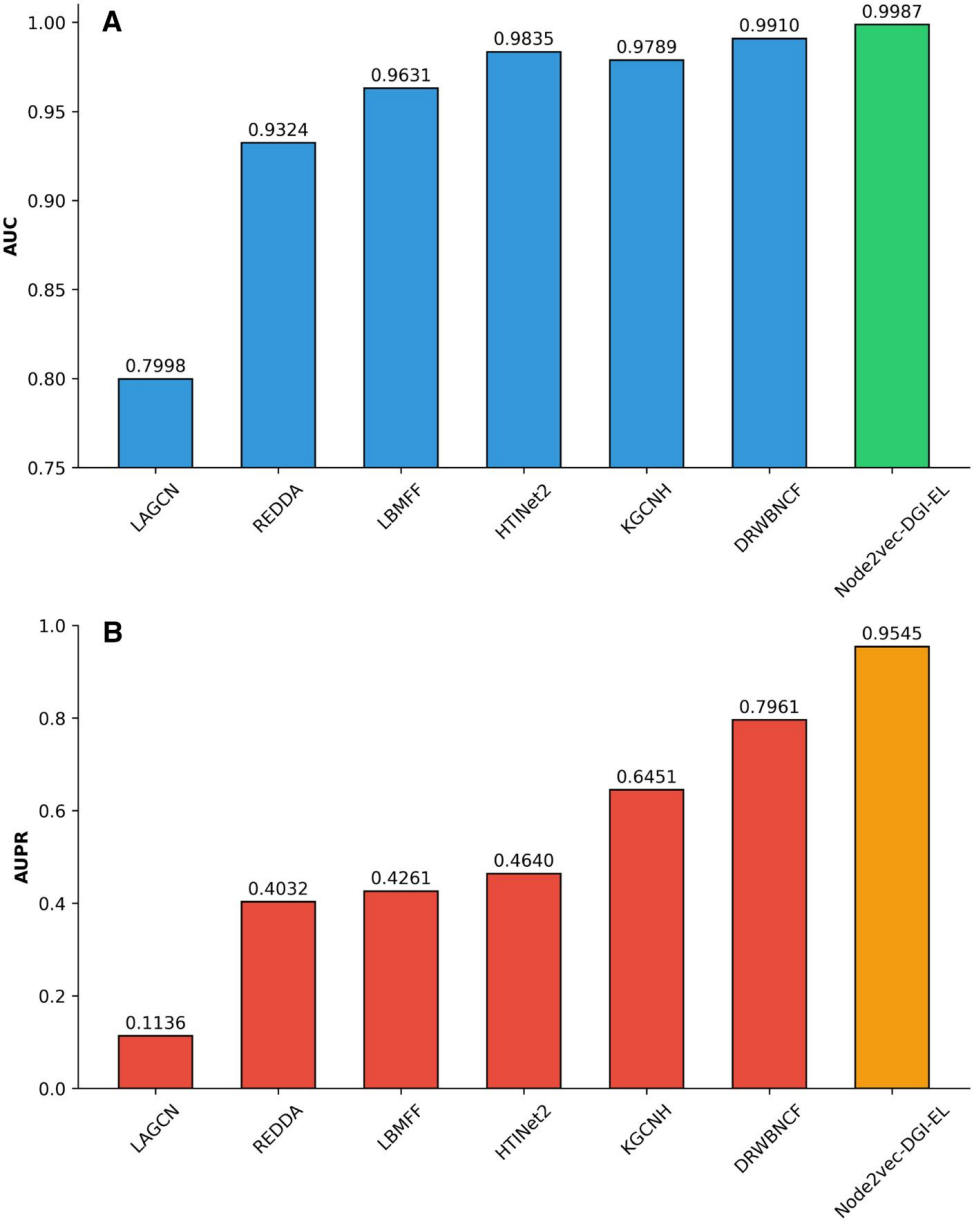
AUC and AUPR of seven methods.

This article compares the proposed Node2Vec-DGI-EL with several benchmark algorithms, including LAGCN, LBMFF, DRWBNCF (Meng *et al.* 2022), REDDA (Gu *et al.* 2022), KGCNH (Du *et al.* 2024), and HTINet2. The experimental results are presented in Table 5. The results show that the Node2Vec-DGI-EL outperforms the other benchmark algorithms across multiple evaluation metrics. Specifically, the AUC reaches 0.9987 (Fig. 3A), and the AUPR reaches 0.9545 (Fig. 3B). Compared to the second-best model (DRWBNCF), our method achieves an improvement of 0.77% in AUC and 15.84% in AUPR. LAGCN achieves an AUC of only 0.7998 and an AUPR of 0.1136, highlighting its limitations for this task. Models such as DRWBNCF and KGCNH also achieve good performance in AUC and AUPR but still show a certain gap compared to Node2Vec-DGI-EL. In summary, the Node2Vec-DGI-EL combines the graph embedding capability of Node2Vec with the representation learning advantage of the DGI framework, enabling it to effectively capture latent information in the data and exhibit superior predictive performance.

**Table 5.** Performance compared with six baseline methods.

Model	AUC	AUPR	ACC	Precision	Recall	F1
LAGCN	0.7998	0.1136	0.7104	0.5265	0.7275	0.4709
REDDA	0.9324	0.4032	0.8948	0.5637	0.8763	0.6019
LBMFF	0.9631	0.4261	0.9241	0.5992	0.9114	0.6439
HTINet2	0.9835	0.4640	0.9186	0.5969	0.9253	0.6399
KGCNH	0.9790	0.7159	0.9427	0.6315	0.9384	0.6524
DRWBNCF	0.9910	0.7961	0.9508	0.6449	0.9577	0.7105
Node2Vec-DGI-EL	0.9987	0.9545	0.9827	0.7716	0.9835	0.8458

### 3.4 Ablation studies

This section designs three ablation models to verify the effectiveness of each module in the Node2Vec-DGI-EL model: (i) Node2Vec-EL, which removes the DGI; (ii) DGI-EL, which removes the Node2Vec and uses the node’s one-hot encoding as the initial feature; (iii) Node2Vec-DGI-LR, which removes the EL and uses a single Logistic Regression classifier; (iv) Node2Vec-DGI-XGBoost, which removes the EL and uses a single XGBoost classifier; (v) Node2Vec-DGI-EL, the complete model architecture. The experimental results are shown in Table 6. The AUPR of the complete model architecture is 0.9545, which is significantly higher than that of the other variants. The AUPR of Node2Vec-EL is 0.7833, with a performance decrease of 17.12%, indicating that the DGI module effectively improves the differentiation of node representations through unsupervised contrastive learning. The AUPR of DGI-EL is 0.8484, with a performance decrease of 10.16%, indicating that the node feature information extracted by Node2Vec enhances the representational capacity of the DGI.

**Table 6.** Comparison of ablation test results.

Model	AUC	AUPR	ACC	Precision	Recall	F1
Node2Vec-EL	0.9867	0.7833	0.9357	0.6167	0.9406	0.6710
DGI-EL	0.9943	0.8484	0.9581	0.6624	0.9659	0.7330
Node2Vec-DGI-LR	0.9748	0.4152	0.9100	0.5883	0.9268	0.6264
Node2Vec-DGI-XGBoost	0.9909	0.7600	0.9419	0.6284	0.9571	0.6884
Node2Vec-DGI-EL	0.9987	0.9545	0.9827	0.7716	0.9835	0.8458

### 3.5 Robustness analysis

**Figure 4.**
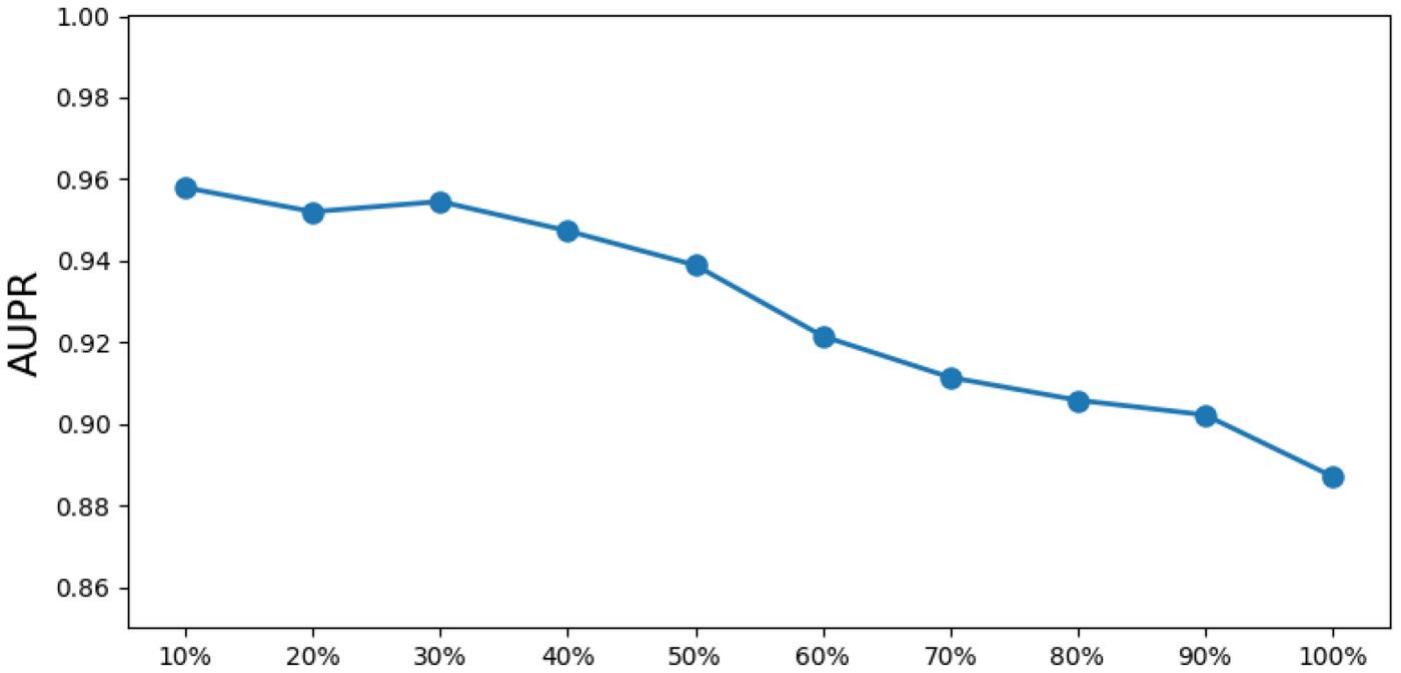
Model AUPR at different levels of data loss.

To evaluate the robustness of the Node2Vec-DGI-EL model, we simulated varying degrees of data loss by randomly hiding edges between ingredients and diseases at different proportions (ranging from 10% to 100%) and analyzed the model’s performance under these conditions. The experimental results are shown in Fig. 4. As the proportion of hidden edges gradually increases, the information that the model can obtain between ingredients and diseases decreases, leading to a gradual decline in the AUPR value. However, the model’s AUPR value remains above 0.88, demonstrating that the Node2Vec-DGI-EL model can still effectively maintain high prediction accuracy and demonstrate strong robustness when facing varying levels of data loss.

### 3.6 Case analysis

This study analyzes two perspectives: predicting diseases highly correlated with ingredients and predicting ingredients highly correlated with diseases, in order to comprehensively validate the reliability of the model’s predictions.

#### 3.6.1 Ingredient–disease association prediction

Triptonide (PubChem CID: 65411) was selected as an example. Based on the model’s predicted values, this ingredient was ranked in descending order with diseases that have no known association in the network, as shown in Table 7. Literature reviews have shown that triptonide can improve streptozotocin-induced diabetic retinopathy in rats (Govindasamy *et al.* 2023), and that low-dose triptonide can effectively protect retinal cells from oxidative damage and inflammation (Li *et al.* 2024), suggesting a potential relationship between triptonide and hypertensive retinopathy. However, no studies have shown that triptonide is associated with the other four diseases, whereas triptolide, which shares a high structural similarity with triptonide (Song *et al.* 2023), is linked to these diseases.

**Table 7.** Triptonide and the predicted top five diseases.

Ingredient	Disease	Score
	Hypertensive retinopathy	0.995
	Hypertensive disease	0.994
Triptonide	Insulin resistance	0.992
	Accelerated skeletal maturation	0.986
	Acanthosis nigricans	0.986

**Figure 5.**
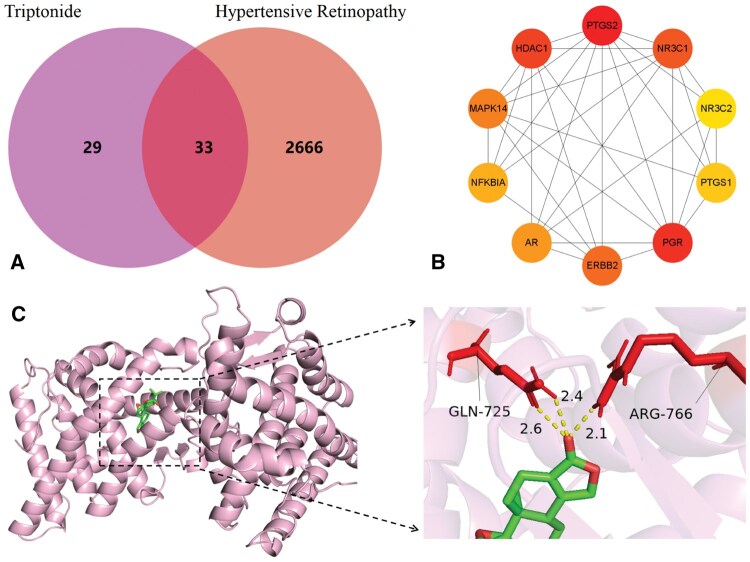
Analysis results of triptonide and hypertensive retinopathy.

This study further explores the relationship between triptonide and hypertensive retinopathy. First, the action targets of triptonide and the disease targets for hypertensive retinopathy are identified, with 33 common targets being obtained through their intersection (Fig. 5A). We constructed a PPI network using STRING and performed visualization analysis with Cytoscape. The core targets were prioritized by the MCC algorithm, revealing the top 10 hub targets (Fig. 5B). Subsequently, Triptonide was docked with the top five core target molecules using AutoDock to identify the optimal binding conformation and calculate molecular binding energy (Table 8). The results indicate that the molecular binding energy between triptonide and PGR is −9.62 kcal/mol, suggesting a stable interaction between the two. The visualization results are shown in Fig. 5C.

**Table 8.** Molecular docking results.

Target protein	PTGS2	PGR	HDAC1	NR3C1	ERBB2
Binding energy	−7.00	−9.62	−5.45	−6.06	−7.57

#### 3.6.2 Disease–ingredient association prediction

**Figure 6.**
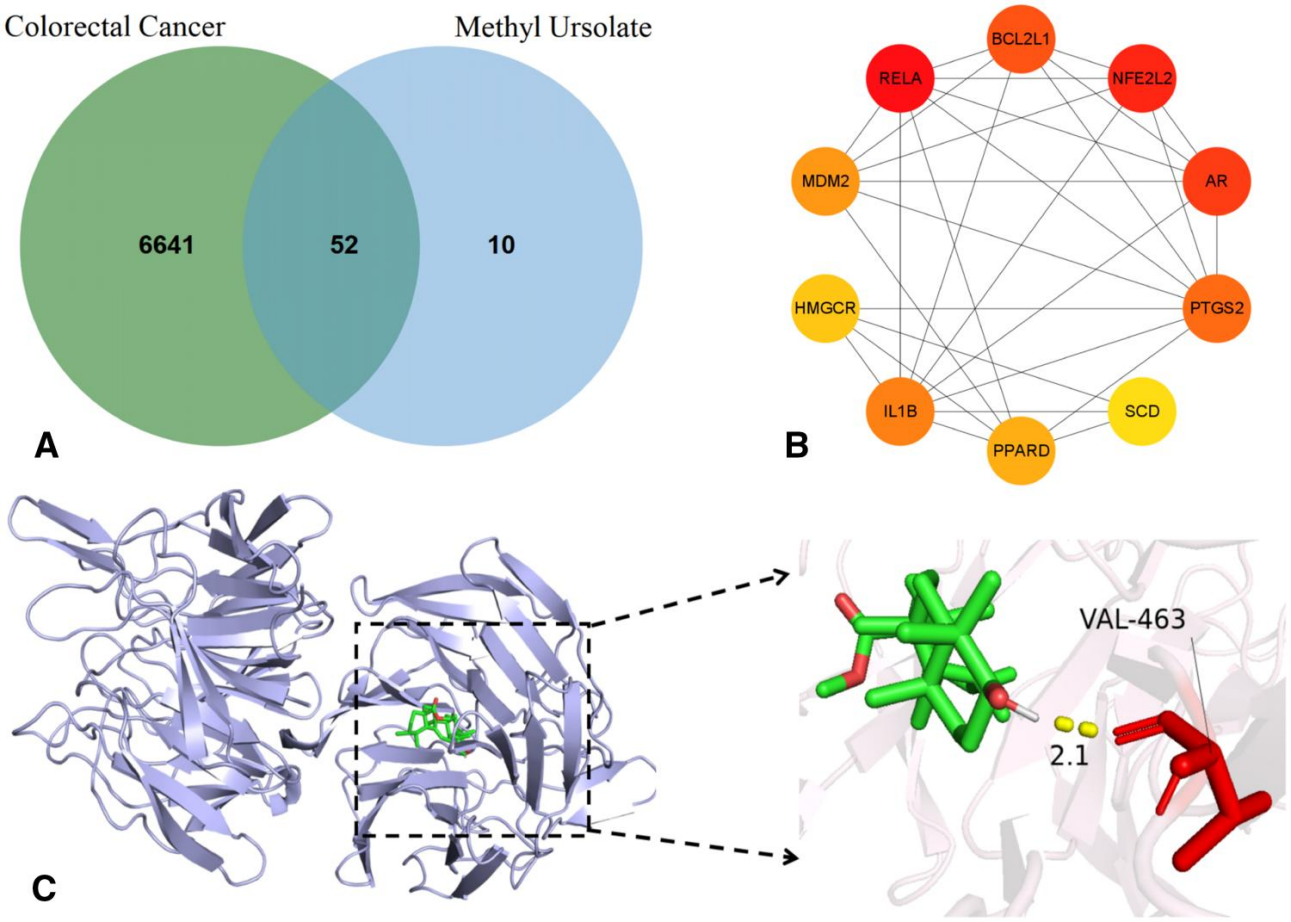
Analysis results of colorectal cancer and methyl ursolate.

This section presents colorectal cancer (CRC) as a case study. The model predicted a high association score (0.9966) between CRC and methyl ursolate (PubChem CID: 636516), suggesting significant pharmacological potential. We identify 52 shared targets through target intersection analysis (Fig. 6A). We constructed a PPI network using STRING and performed visualization analysis with Cytoscape. The core targets were prioritized by the MCC algorithm, revealing the top 10 hub targets (Fig. 6B).Molecular docking with AutoDock shows NFE2L2 has the strongest binding affinity (−9.71 kcal/mol) with methyl ursolate (Table 9, Fig. 6C), indicates stable ligand–receptor interaction.

**Table 9.** Molecular docking results.

Target protein	RELA	NFE2L2	AR	BCL2L1	PTGS2
Binding energy	−6.88	−9.71	−7.85	−6.85	−6.91

## 4. Discussion

TCM ingredients represent a vital source for drug discovery, serving as both the foundation for traditional drug development and a critical resource for modern pharmaceutical innovation. In this study, we first evaluated the predictive performance of five classical graph representation learning algorithms alongside the DGI on the HITD network. Notably, the Node2Vec-DGI-EL model outperformed all comparator methods across multiple evaluation metrics. Furthermore, hyperparameter optimization yielded an optimal parameter combination, achieving exceptional predictive performance (AUC = 0.9987, AUPR = 0.9545). Importantly, ablation experiments demonstrated that the synergistic interaction between Node2Vec and DGI modules is pivotal to model enhancement, suggesting these methods complement each other’s limitations and collectively improve generalization. Finally, robustness analysis confirmed the model’s reliability under varying degrees of data sparsity.

To further validate the model’s efficacy, we conducted case studies on triptonide and methyl ursolate, investigating their potential therapeutic roles in hypertensive retinopathy and CRC, respectively. For triptonide, our predictions identified hypertensive retinopathy as its highest-scoring association, with molecular docking revealing strong binding (ΔG=−9.62 kcal/mol) to PGR, a core target in this condition. Given that triptonide exhibits broad pharmacological activities (e.g. anti-inflammatory, anticancer, and immunomodulatory effects) (Song *et al.* 2023) and PGR mediates neuroprotection and ocular blood flow regulation (Nuzzi *et al.* 2018), our findings suggest triptonide may mitigate hypertensive retinopathy by targeting PGR, offering novel therapeutic insights. Similarly, methyl ursolate showed the highest predicted association with CRC, binding robustly (ΔG=−9.71 kcal/mol) to NFE2L2 (NRF2), a key regulator of oxidative stress (Pajares *et al.* 2016). Although NFE2L2 activation protects normal cells, its hyperactivation in tumors promotes proliferation and drug resistance (Rojo de la Vega *et al.* 2018). Thus, methyl ursolate’s known anticancer properties (Sultana 2011, Ramadwa *et al.* 2017) may arise from NFE2L2-mediated modulation of oxidative stress pathways, potentially inhibiting tumor progression.

However, several limitations warrant consideration. First, the HITD network relies primarily on ETCM and STRING, whose limited coverage may constrain the model’s predictive accuracy. Future studies should integrate additional TCM-related data to enhance network comprehensiveness. Second, while DGI demonstrated competitive performance, alternative contrastive learning algorithms may further optimize representation learning. Lastly, although molecular docking provided preliminary validation, *in vitro* (e.g. cell-based assays) and *in vivo* (e.g. animal models) experiments are essential to fully assess the model’s translational potential.

## 5 Conclusion

The Node2Vec-DGI-EL model proposed in this study integrated three computational approaches: the Node2Vec for graph representation learning, the DGI for depth graph embedding, and ensemble learning methods. By applying this framework to the HITD network, we systematically explored potential associations between ingredients and diseases, consequently enabling the identification of highly relevant ingredient–disease pairs. This integrative approach offers significant advantages for TCM research. Node2Vec-DGI-EL was improved based on the characteristics of TCM, particularly enhancing its ability to reveal hidden relationships and accelerating the modernization of TCM.

## Data Availability

The code and data are available at https://github.com/wayfarer569/Node2Vec-DGI-EL.
